# Mixed-Effects Modeling of Neurofeedback Self-Regulation Performance: Moderators for Learning in Children with ADHD

**DOI:** 10.1155/2018/2464310

**Published:** 2018-03-22

**Authors:** Agnieszka Zuberer, Franziska Minder, Daniel Brandeis, Renate Drechsler

**Affiliations:** ^1^Department of Child and Adolescent Psychiatry and Psychotherapy, University Hospital of Psychiatry Zurich, University of Zurich, Zurich, Switzerland; ^2^Neuroscience Center Zurich, University of Zurich and ETH Zurich, Zurich, Switzerland; ^3^Department of Child and Adolescent Psychiatry and Psychotherapy, Central Institute of Mental Health, Medical Faculty Mannheim/Heidelberg University, Mannheim, Germany; ^4^Center for Integrative Human Physiology, University of Zurich, Zurich, Switzerland

## Abstract

**Introduction:**

Neurofeedback (NF) has gained increasing popularity as a training method for children and adults with attention deficit hyperactivity disorder (ADHD). However, it is unclear to what extent children learn to regulate their brain activity and in what way NF learning may be affected by subject- and treatment-related factors.

**Methods:**

In total, 48 subjects with ADHD (age 8.5–16.5 years; 16 subjects on methylphenidate (MPH)) underwent 15 double training sessions of NF in either a clinical or a school setting. Four mixed-effects models were employed to analyze learning: training within-sessions, across-sessions, with continuous feedback, and with transfer in which performance feedback is delayed.

**Results:**

Age and MPH affected the NF performance in all models. Cross-session learning in the feedback condition was mainly moderated by age and MPH, whereas NF learning in the transfer condition was mainly boosted by MPH. Apart from IQ and task types, other subject-related or treatment-related effects were unrelated to NF learning.

**Conclusion:**

This first study analyzing moderators of NF learning in ADHD with a mixed-effects modeling approach shows that NF performance is moderated differentially by effects of age and MPH depending on the training task and time window. Future studies may benefit from using this approach to analyze NF learning and NF specificity. The trial name Neurofeedback and Computerized Cognitive Training in Different Settings for Children and Adolescents With ADHD is registered with NCT02358941.

## 1. Introduction

Neurofeedback (NF) is a training method by which real-time feedback of brain activity, typically an EEG parameter, is delivered to the subject to promote voluntary control of brain activity. The subject has electrodes attached to the head, and the measured EEG parameter is converted to a sound or visual stimulus, which is then fed back to the subject. The main NF protocols for patients with attention deficit hyperactivity disorder (ADHD) are the training of frequency bands and the training of slow cortical potentials (SCPs). Frequency band NF targets tonic aspects of activation by promoting learning to reduce or to enhance activity of defined frequency bands. SCP training targets the phasic regulation of cortical excitability by learning to generate negative and positive shifts of cortical activity. SCPs originate in the apical dendritic layers of the neocortex and reflect synchronized depolarization of large groups of neuronal assemblies. According to Birbaumer's threshold regulation model of cortical excitation [1], negative and positive SCPs are associated with an activated (i.e., more attentive) or deactivated (less attentive/more relaxed) state, respectively.

Although frequency band training is the most common form of NF for ADHD, recent research no longer supports the presumption that increases in theta power, reductions in beta power, or the theta/beta ratio is a reliable ADHD marker and, in consequence, compelling targets for NF [2–4]. A rationale for using SCP NF is the relatively robust finding of an ADHD-related reduction of the contingent negative variation (CNV), a SCP which reflects preparation and activation and has been shown to normalize partially after SPC-NF training (e.g., 5 and 6). In addition, regulation rather than normalization may be the target of the training [5].

In recent meta-analyses of NF efficacy for ADHD [6, 7], significant treatment effects were found for parents' but not for teachers' ratings. Teacher ratings are regarded as a more valid measure for treatment effects as they are probably blinded to what type of training was administered. These reviews did not consider whether subjects showed successful NF learning; however, this is an important aspect of training specificity. If children show good clinical improvements without successful NF learning, changes have to result from other nonspecific aspects of the training. NF learning denotes the ability to modulate the NF target parameter across multiple sessions. Thus, we will refer to the ability to modulate the NF parameter as “NF learning” without presumptions about its efficacy (in line with e.g., 10 and 11). The few studies that have examined NF learning across the course of the training differed considerably in their methodological approaches and definition of learner rates (see 12, for a review). In addition, it has been argued that the analysis of within-session learning across the training course would result in a more robust measure than analyzing cross-session learning alone. Through averaging multiple sessions, the measurement overall error variance would be reduced [8]. From a clinical perspective, such within-session analysis also allows progressive fatigue effects towards the end of a session to be controlled for.

The transfer of learning in NF with respect to everyday life situations is hypothesized to be better practiced in the transfer condition than in the feedback condition. In the transfer condition, the subject has to modulate the NF parameter without the aid of a feedback stimulus. The transfer condition is hypothesized to be closer to everyday life situations as compared to the feedback condition, where continuous performance feedback is available [9]. However, only few studies report results on that type of condition [9–12].

Neurofeedback for ADHD has mainly been perceived as an alternative for stimulant medication, but the combined effects of medication on NF learning are unknown. In several ADHD NF studies, MPH has been permitted in constant dose [10, 12–15] or without explicit restrictions [9, 16, 17], whereas in other studies, it has been an exclusion criterion [18, 19]. Moreover, the few studies that included medication effects in their analyses did not consider their impact on NF learning [9, 20, 21]. There is little evidence on how stimulants might affect NF learning in ADHD [22–25]. In other clinical intervention studies, it has been shown that behavioral therapy resulted in stronger clinical outcome improvements when combined with MPH as compared to receiving therapy only [26, 27]. However, the impact of MPH on learning progress in training studies in ADHD has been barely examined.

Although a great deal of evidence suggests that EEG activity is associated with age [28–30], to our knowledge, it has barely been employed as a possible covariate for NF learning [31]. It is also unknown whether contextual and administration factors, such as intensity and duration of sessions, training location, and context—for instance at school, in a summer camp, or in a clinical setting—may systematically alter the ability to regulate one's brain activity. A precise description of NF learning is necessary to get a better understanding, whether at all, and if at all, by what degree and in which form learning does take place. This question is vital since training progress may be a necessary condition to induce clinical improvements and plastic neuronal changes, at least in a sizable proportion of children [32]. However, most NF studies in ADHD looked at pre- and post changes, leaving out the question of learning.

One challenge in analyzing training studies across multiple sessions is that the training performance variability varies considerably not only across time within a single subject but also across multiple subjects, which compromises conventional basic statistical methods, where correlations between observations are often obstructive. For that reason, we opted for a mixed-effects modeling approach. One major advantage of mixed-effects modeling is that it does not assume independence among observations and is to some degree more robust with unbalanced data than basic multivariate analysis (36).

In this study, we analyze NF learning in children and adolescents with ADHD. The major research question of this paper is (1) whether, and to what degree, both subject-specific (e.g., age or IQ) and treatment-related factors (e.g., school versus clinical treatment setting) may be related to NF learning within and across sessions, (2) whether NF learning differs in feedback and transfer conditions, and (3) whether within-session analysis can contribute additional information to cross-session analysis.

## 2. Methods and Materials

### 2.1. Participants

Subjects were recruited in outpatient clinics, by referral of clinicians, in parent self-aid groups, and at schools. Forty-four subjects, of whom 33 had a clinical ADHD diagnosis before entering the study, were included. See Table 1 for group characteristics.

Inclusion in the study required written consent by both the child and parents. The study was approved by the local ethics committee. Age ranged from 8.5 to 16.5 years. Inclusion in the study was based on clinically relevant scores in the German version of the Conners 3 parent and Conners 3 teacher rating scales [33], according to DSM-IV criteria (one of two ADHD DSM-IV indices reaching *T* values ≥65, the other *T* ≥ 60 according to both teachers' and parents' ratings for children of the combined subtype; ADHD DSM-IV inattention *T* ≥ 65 in one and *T* ≥ 60 in the rating for the inattentive subtype).

Medication with methylphenidate (MPH) was allowed if the dose was kept stable over the full treatment time, including three months before the first assessment. For children taking MPH, teacher and parent ratings had to be based on the behaviour on medication. Exclusion criteria were estimated IQ ≤ 80 (short form of the German WISC-IV [34]), taking atomoxetine or a neuroleptic or other psychoactive drug, severe comorbidities or other psychiatric disorders, neurological disorders, previous experience with NF (more than four lessons), or either participating in or planning to start a treatment which might confound training effects. Sufficient knowledge of the German language was a further precondition so as to fully understand instructions (children) or to complete questionnaires (parents). Parents had to complete the Development and Well-Being Assessment ((DAWBA) [35]) to screen for comorbid clinical conditions.

### 2.2. Study Design

**Figure 1:**
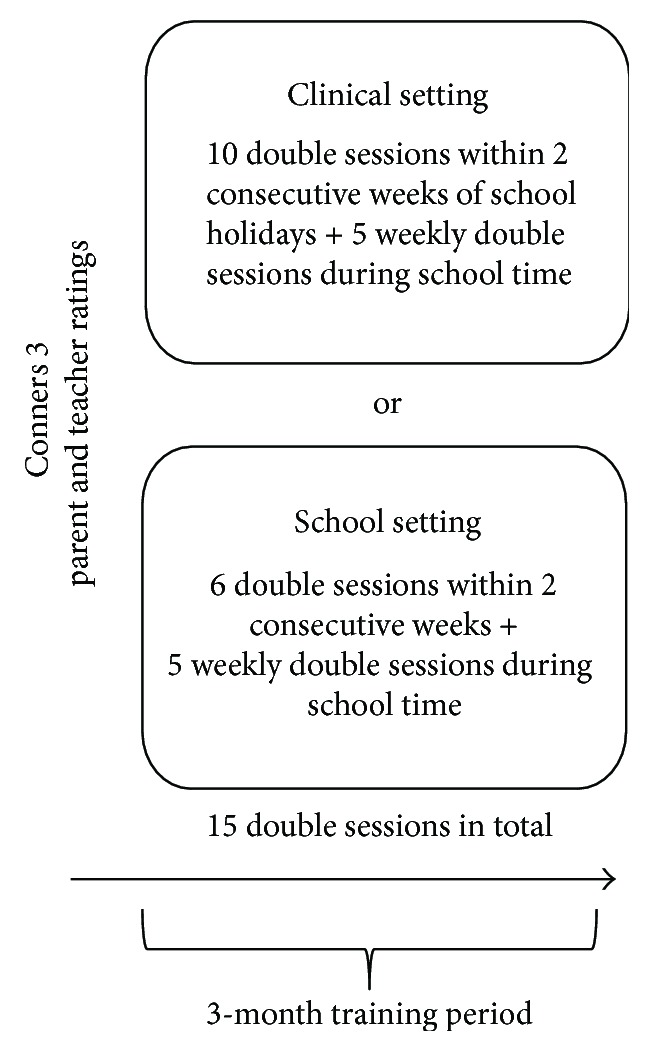
Study design.

Parents and teachers rated the child's behaviour on the Conners 3 scales and the Behaviour Rating Inventory of Executive Function (BRIEF) [36] before training onset. This study focusses on the NF treatment phase of a larger project that involved additional assessments and another treatment group. Their specifications are not relevant for the present analyses and are described elsewhere [37]. About half of the children (*N* = 23) underwent NF training in the outpatient clinic of the Department of Child and Adolescent Psychiatry (clinical setting). The other children (*N* = 21) were trained at school in a separate room, during normal school hours (school setting). A complete training comprised 15 double sessions (approximately 100 min) administered over 10 to 12 weeks. The actual training took around 60 minutes, around 30 minutes per session. The rest of the time was needed to attach and deattach the electrodes on the child's scalp, to complete short questionnaires on well-being and motivation, and to check for transfer and for a short break between sessions, sometimes with refreshments or a snack. In the clinical setting, training started as a 2-week vacation course with double training sessions daily (five double sessions per week; see Figure 1) followed by weekly double sessions over at least five weeks. The relatively intensive format for the first training phase was chosen to ensure the consolidation of learning in NF, whereas the last 5 double sessions were regarded as freshen up sessions. As the training in the school setting did not allow five training sessions a week (due to losing too many classes in a row), the training frequency in the intensive phase was kept on 2-3 double sessions per week. A maximal break of 10 days was permitted during the last training phase (e.g., during vacation). In the school setting, two to three sessions per week were administered for the first two weeks, followed by one weekly session over at least seven weeks (see Figure 1). Training in the school setting was administered during the school lesson time in a separate room.

### 2.3. Description of the NF Training

**Figure 2:**
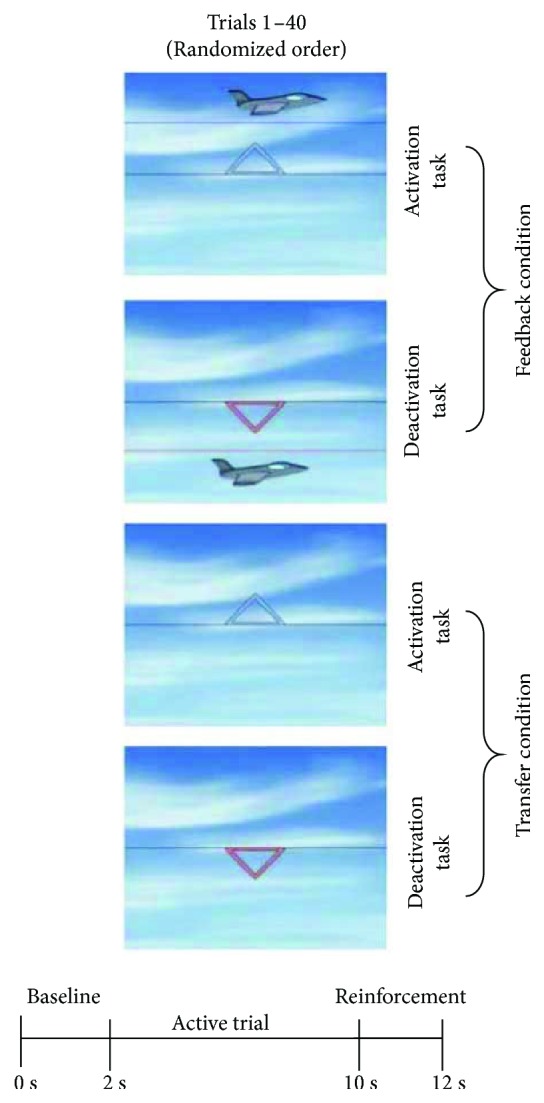
Setup of the SCP-NF. Feedback/transfer condition: condition where a feedback stimulus is (feedback) or is not (transfer) visible. Deactivation task: generation of positive potential shifts. Activation task: generation of negative potential shifts. 1 double session consists of 4 blocks with 40 trials each, each block including feedback and transfer conditions and deactivation/activation tasks as illustrated (pictures by Ilmenau, neuroConn GmbH).

NF was provided using a commercially available mobile training device (THERA PRAX; neuroConn GmbH). Double sessions consisted of four blocks, each containing 40 trials (see Figure 2). The subject was seated in a comfortable chair in front of a computer monitor. The NF training was presented as a computer game. Depending on the colour and direction of a centrally fixated triangle, the subject was instructed to either activate (produce negative SCP shifts; red upwards-pointing triangle) or deactivate (produce positive SCP shifts; blue downward-pointing triangle). One SCP trial lasted 12 seconds and consisted three phases (see Figure 2): a baseline phase (seconds 2 s), an active phase (8 s), and a reinforcement phase (2 s). In the feedback condition, a direct feedback stimulus appeared, while in the transfer condition, no feedback stimulus was provided. In the feedback condition, the subject was instructed to steer a stimulus (e.g., fish and airplane) above or below a central horizontal line while it moved from left to right across the screen. The change in activation was fed back by the target stimulus, whose vertical position was proportional to the SCP shift. Good performance (stimulus was kept at least two seconds above or below a predefined threshold of ±40 *μ*v) was rewarded in both conditions by a reward stimulus (sun) at the end of the trial. All conditions (feedback/transfer) and tasks (activation/deactivation) appeared in randomized order (after conditions and tasks “feedback/transfer,” “activation/deactivation”; see Figure 2). The proportion of activation and deactivation trials was always equal in each block (50% each). The percentage of transfer trials increased gradually with session and block number, as it was expected that with increasing training experience the acquired skill would be transferred to trials where no concomitant visible feedback was provided (“transfer condition”). Transfer trials per training block of a single double session were administered in the following percentages: double sessions 1-2: 20/20/20/20, double sessions 3-5: 20/20/20/40, double sessions 6-8: 20/20/40/40, double sessions 9-13: 20/40/40/50, and sessions 14-15: 50/50/50/50.

### 2.4. Montage and EEG Recording

The participants' EEGs were recorded at electrode Cz, referenced to the right mastoid electrode (ground was left mastoid) shunted over a 10 kOhm resistance (impedance < 20 kOhm; sampling rate was 512 Hz). The EEG amplifier (THERA PRAX, neuroConn©) used a low-pass filter of 40 Hz. Filtering of the SCPs was performed from 0.01–40 Hz with a two-way least-squares FIR filter. Preprocessing was performed with MATLAB and EEGLAB. Processing of the SCPs (DC—2 Hz) was performed from channel Cz-A2 for each sample point and displayed on the trainer screen. The maximal time delay until the patient saw the feedback of the NF parameter was about 110 ms. Display of the change in mean amplitude with respect to the pretrial baseline was fed back by the vertical movement of the feedback stimulus, whereas its horizontal position corresponded to the time axis. Trials were baseline corrected (the mean amplitude of the pretrial baseline was subtracted from each data point of the SCP amplitude) and then averaged. Since we frequently observed muscle activity in the first second of the trial, we only incorporated the last 6 seconds of the recording in the active trial. As regression-based artefact correction procedures did not yield reliable results, we applied a strict artefact removal procedure, where after manual artefact rejection, baseline-corrected trials were rejected if their amplitudes exceeded ±100 mV or their gradients exceeded 50 mV between two data points.

### 2.5. Statistical Analysis

Four separate models were analysed to predict performance in the feedback condition and transfer condition either across or within sessions. Statistical analysis was performed with a linear mixed-effects (LME) regression [38] following a step-up approach, where a random effect was retained if there was a significant difference between the log-likelihood ratio of a model that contained the random effect and a model that did not (as compared with ANOVA; *p* < 0.05). Following the principle of marginality, main effects for higher-order interactions were kept in the model [39]. To control for high type I error rate inflation, we also included a random slope coefficient in the model [40, 41]. Statistical analysis was performed using the lme4 package in R [42]. Models to predict NF learning with respect to within-/cross-session learning and type of condition (feedback and transfer) were analysed in separate models to prevent possible overparametrization. The dependent variable was mean amplitude (*μ*V). For cross-session analysis, the mean amplitude of each baseline-corrected trial was averaged for each session. For within-session analysis, the mean amplitude of each baseline-corrected trial was averaged across sessions and then further averaged across 10 equally spaced units (from here on called bins). All analyzed effects are summarized in Table 2 and ANOVA tables in supplementary S3 and S4 for feedback and transfer condition, respectively.

## 3. Results

### 3.1. Feedback Condition

The statistics of the best model fit for each of the four models to predict NF performance are presented in the following sections. We will call performance progress in each condition “feedback learning” and “transfer learning,” respectively.

#### 3.1.1. Cross-Session Feedback Learning

**Figure 3:**
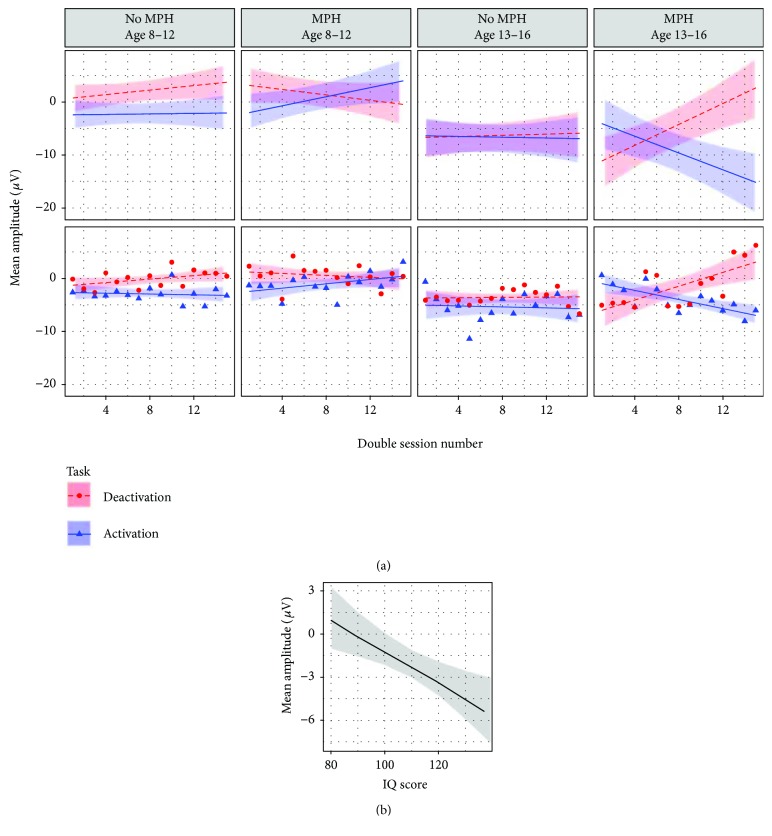
Visualization of effects moderating cross-session NF learning in the feedback condition. The dependent variable is mean amplitude (*μ*V) of baseline-corrected trials. (a) Interaction effect between session, task, age, and MPH. For comparison between effects and raw data, see scatter plot under each effects panel, fitted with a least squares regression based on the same factors as in the effect plots. Session number: 15 training sessions in total. Task. Deactivation: generation of positive potential shifts. Activation: generation of negative potential shifts. MPH: being on constant methylphenidate medication. Feedback condition: feedback stimulus visible. For visualization, age is subdivided into two age classes (8–12 and 13–16 years), but preserved as a continuous variable in the original model. (b) Visualization of IQ effect. IQ is estimated based on the short form of the German WISC-IV [34].

As shown in Table 3, the final model for cross-session learning for the feedback condition included subject as random intercept (*τ*_00_ = 7.214) and session number as random slope (*τ*_11_ = 0.0734). As shown in Figure 3(a), a four-way interaction between session number, task, age, and MPH resulted in the best model fit (*β* = 0.32; CI = 0.16–0.47; *p* < 0.001). As shown in Figure 3(b), IQ was negatively associated with mean amplitude (*β* = −0.08; CI = −0.14 to −0.02; *p* = 0.006), meaning that with increased IQ a more negative mean amplitude occurred. The inclusion of the remaining effects summarized in Table 2 did not result in a better model fit.

As shown in Figure 3(a), the desired learning pattern, showing a positive slope in the deactivation task and a negative slope in the activation task, became more prominent with increasing age and MPH. To test for possible overparametrization effects due to the complex four-way interaction, a separate model was analyzed in which the task effect was omitted and accounted for in the dependent variable: The dependent variable was the SCP differentiation, the difference between mean amplitudes of deactivation, and activation. The results are in line with the original model (see Figure S1 and Table S2). ANOVA results for the feedback learning models are shown in the supplement (Table S3 and Table S4).

#### 3.1.2. Within-Session Feedback Learning

**Figure 4:**
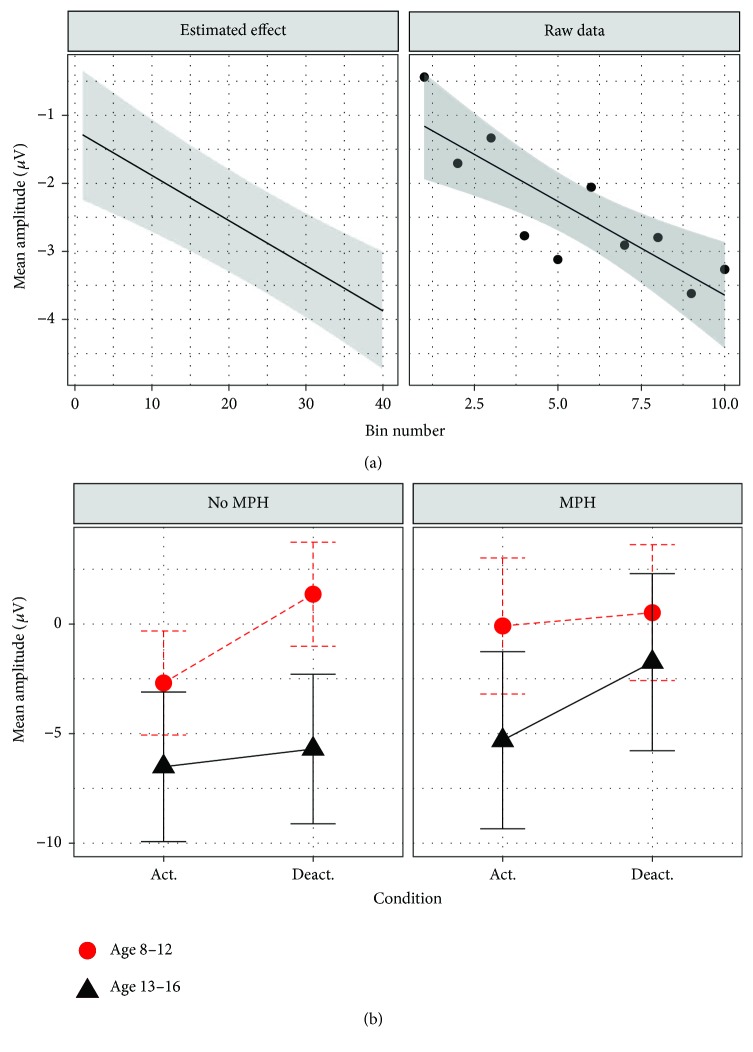
Visualization of effects moderating within-session NF learning in the feedback condition. The dependent variable is mean amplitude (*μ*V) of baseline-corrected trials. (a) Effect plot for the effect bin number. For comparison between effect and raw data, see scatter plot in the right panel. Bin number: trials of all sessions were averaged and subdivided into ten equally spaced units. For visualization age is subdivided into two age classes (8–12 and 13–16 years), but preserved as a continuous variable in the original model. (b) Effect plot for the interaction between MPH, age, and task. Act.: activation task. Deact.: deactivation task.

The final model for within-session learning for the feedback condition included subject as random intercept (*τ*_00_ = 9.093) and bin number as random slope (*τ*_11_ = 0.05569). As shown in Figure 4(a), an increasing bin number was associated with a more negative mean amplitude (bins: *β* = −0.29; CI = −0.40 to −0.18; *p* < 0.001). Thus, over the course of a session, subjects managed to generate more negative potentials, irrespective of the condition. A higher IQ was associated with a more negative mean amplitude (*β* = −0.07; CI = −0.13 to −0.01; *p* = 0.018) and was comparable to the effect achieved in the cross-session model for FB learning (see Figure 3(b)).

A three-way interaction between task, MPH, and age resulted in the best model fit (*β* = 0.86; CI = 0.36–1.35; *p* = 0.001; see Figure 4(b)). The ability to regulate (activation and deactivation) in the desired direction was positively associated with both MPH and age: The inclusion of effects of the remaining factors summarized in Table 2 did not result in a better model fit.

### 3.2. Transfer Condition

#### 3.2.1. Cross-Session Transfer Learning

**Figure 5:**
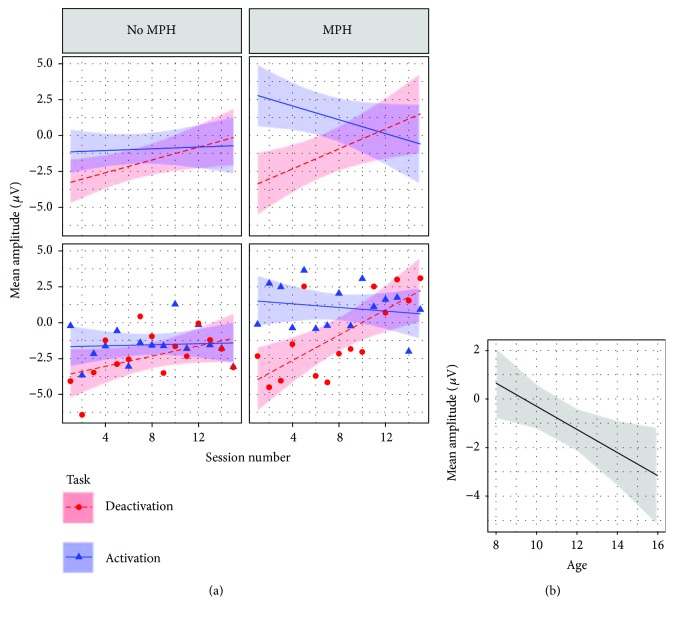
Visualization of effects moderating cross-session NF learning in the transfer condition. (a) Interaction effect between session, task, and MPH. Transfer condition: no continuous feedback stimulus visible. Task. Deactivation: generation of positive potential shifts. Activation: generation of negative potential shifts. MPH: being on constant methylphenidate medication. (b) Age effect plot.

The final model for cross-session learning for the transfer condition included subject as random intercept (*τ*_00_ = 8.501) and session number as random slope (*τ*_11_ = 0.1325; Table 3). A three-way interaction between the fixed effects session number, task, and MPH resulted in the best model fit (*β* = 0.39; CI = 0.05–0.76; *p* = 0.036). As shown in Figure 5(a), performance improved predominantly in the deactivation task, while remaining stable in the activation task. MPH was associated with larger performance increments in the deactivation task as compared to no MPH. As shown in Figure 4(b), age was negatively associated with amplitude (*β* = −0.59; CI = −0.94 to −0.24; *p* = 0.002). Thus, NF learning was rather prominent when being on constant methylphenidate medication. The inclusion of effects of the remaining factors summarized in Table 2 did not result in a better model fit.

#### 3.2.2. Within-Session Transfer Learning

**Figure 6:**
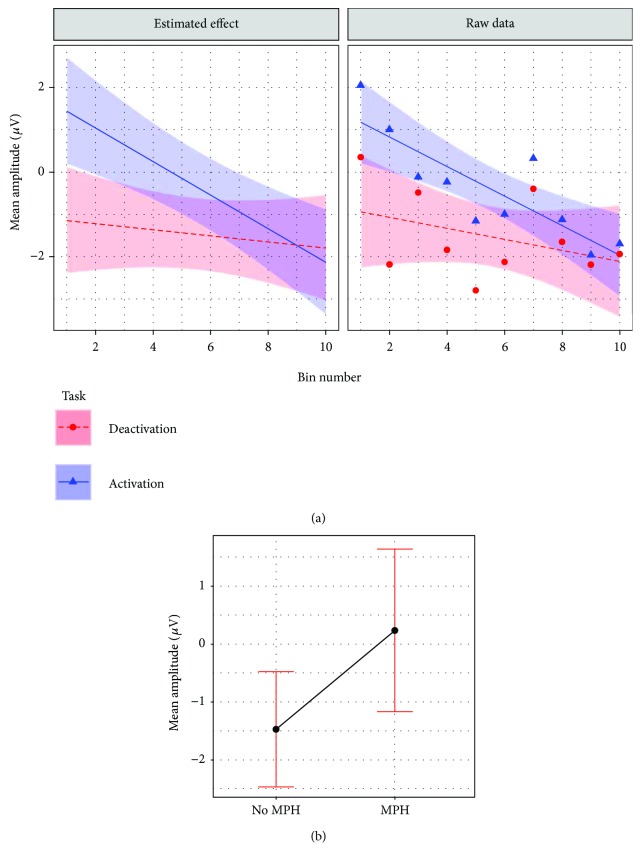
Visualization of effects moderating within-session NF learning in the transfer condition. (a) Interaction effect between bin number and task. Transfer condition: no continuous feedback stimulus visible. Task. Deactivation: generation of positive potential shifts. Activation: generation of negative potential shifts. (b) MPH effect plot. MPH: being on constant methylphenidate medication.

The final model for within-session learning in the transfer condition included subject as random intercept (*τ*_00_ = 13.320) and bin number as random slope (*τ*_11_ = 0.1231). As shown in Figure 6(a), a two-way interaction between bin number and task resulted in the best model fit (*β* = 0.33; CI = 0.07–0.56; *p* = 0.011). Age was negatively associated with mean amplitude and was comparable to the effect of the model predicting cross-session learning (see Figure 5(b); *β* = −0.40; CI = −0.78 to −0.00, *p* = 0.040). As shown in Figure 6(b), MPH was positively associated with mean amplitude (*β* = 1.71; CI = 0.03–3.40, *p* = 0.051). ANOVA results of both transfer learning models are shown in the supplement (S4).

Thus, NF learning in the transfer condition took place in the activation task rather than in the deactivation task. Moreover, being on constant methylphenidate medication was associated with a more positive mean amplitude (see Figure 6(b)), while age was negatively associated with mean amplitude. The inclusion of the remaining factors summarized in Table 2 did not result in a better model fit.

### 3.3. Artifacts

We also analyzed whether NF learning was associated with the number of trials rejected due to artifacts by performing separate models for within and cross-session learning that included artifact rejection in the models. The mean artifact rate was 29.1% (±17%). The inclusion of the artifact rate did not yield a significantly better model fit for either condition.

### 3.4. Learning Rates

To explore the number of subjects showing the desired learning slope in cross-session NF learning, models for both the feedback and transfer conditions were calculated separately and the subjects' random slopes were extracted to determine the individual learning performance for each task. Successful NF learning was defined by a negative slope in the activation task or a positive slope in the deactivation task. Subjects presenting both a positive slope in the deactivation task and a negative slope in the activation task were labelled “successful regulators.” In the feedback condition, 20 learners (41.7%) in the activation task, 23 learners (47.9%) in the deactivation task, and 10 subjects (20.8%) were classified as successful regulators. In the transfer condition, 23 subjects (47.9%) were classified as learners in the activation task, 23 as learners in the deactivation task (47.9%), and eight as successful regulators (16.7%).

## 4. Discussion

This paper addresses the lack of NF studies in ADHD that map learning in NF and control for both treatment-related effects, such as setting and time frequency, and subject-related effects, such as IQ and stimulants. It presents the groundwork for measuring treatment specificity [43] by presenting a novel methodological approach, mixed-effects modeling, to investigate learning in NF both across- and within-sessions. Applying mixed-effects modeling enabled us to show that NF learning is indeed moderated by subject-related factors. The moderators partially differ when performance feedback is provided continuously (feedback condition) and delayed (transfer condition) and when within-session or cross-session learning is considered.

### 4.1. Feedback Condition

#### 4.1.1. Cross-Session Feedback Learning

Children on constant MPH showed stronger performance increments across sessions with increasing age (age range between 8.5 and 16.5 years). In contrast, children who did not take MPH showed less pronounced potential shifts than when on constant stimulant medication. For these children, learning was negatively moderated by age, albeit the generation of potential shifts was still in the desired relative direction (mean amplitude in the activation task was more negative than that in the deactivation task).

#### 4.1.2. Within-Session Feedback Learning

Similarly to the cross-session NF model, performance was also interacting with age and MPH of comparable direction and strength. In contrast to cross-session analyses, children generated negative potential shifts within sessions irrespective of task and time. However, the generation of potential shifts remained in the desired direction (mean amplitude in the activation task was more negative than that in the deactivation task). Thus, children produced progressively more negative potential shifts throughout a session, irrespective of whether the task demanded positive or negative potential shifts. Since moderators of learning have been rarely examined in SCP-NF before, these findings are difficult to explain in the context of previous research. It is open to speculation whether this finding might reflect the time required to fully mobilize attentional resources within a session. The added value of within-session analyses in the feedback condition relies here on the possibility that two consecutive training sessions of NF might not necessarily be too tiring for children and adolescents with ADHD; on the contrary, our findings might even indicate that subjects need time to immerse themselves in the training scenario if they are to tap into the full potential of the training, especially with respect to the activation task. Thus, it might even be recommended to perform trainings in the form of double sessions.

#### 4.1.3. General Discussion Feedback Learning

The NF literature offers little help in interpreting these opposite findings with respect to medication and age (feedback learning across sessions was positively associated with age for children with stimulants, but negatively associated with age for medication-free children). Previous NF studies allowing MPH have not included these factors as covariates for learning together [9, 10, 12, 13, 16, 17], although there are few studies considering age as a moderator of frequency band NF learning [44]. It appears that the self-regulation of brain activity in the feedback condition is positively associated with both maturation and intake of stimulants. One possible explanation for this interaction of age and MPH is that substantial performance progress in NF might be dependent on executive functioning (EF), which has been shown to improve with maturation [45] and intake of stimulants [46]. Thus, age-related improvements of EF might have been a necessary but not a sufficient condition to NF learning, with medication being the critical factor for learning with increasing age.

Taken these results together, it appears that feedback learning may become easier and faster with MPH and increasing age. Therefore, it might be more beneficial for older children taking stimulants to increase the proportions of transfer trials earlier in training sessions than for younger children not taking stimulants. Older subjects taking stimulants might benefit earlier from generalizing effects of the acquired NF skills. In contrast, younger children without MPH might need more training sessions and more feedback trials to consolidate the NF skills.

Children with a higher estimated IQ generated more negative potentials, irrespective of other effects such as time, task, age, and stimulants. This finding was expected and is supported by another study showing that the CNV, one form of a SCP reflecting cognitive mobilization, was positively associated with IQ [47]. A general confounding factor might be that children on medication still had to present clinically relevant symptoms to be included in the study. In such cases, consequently, either the medication was ineffective or the clinical impairment would have been more severe if not on MPH. Thus, children on MPH might actually be even more clinically impaired than suggested by behavioral measures. This might further explain why younger children on stimulant medication, being possibly more severely affected than the age-matched nonmedicated children, but without the maturated EF skills of the older medicated children, had more difficulty in learning EEG regulation.

### 4.2. Transfer Condition

#### 4.2.1. Cross-Session Transfer Learning

Transfer learning was especially challenging, as shown by potential shifts that were smaller than those in feedback learning. As no continuous performance feedback is available during the transfer condition, regulating attention becomes more difficult. Furthermore, and in line with Strehl et al. [9] and Drechsler et al. [10], transfer learning was more evident in the deactivation task. This finding cannot be explained by simple cross-session motivation decrements, as these would lead to decreased attention and thus produce a positive learning slope in both conditions. Indeed, it might have been more difficult for the group to improve average performance in the activation task (voluntary upregulation of attention) than in the deactivation task (voluntary downregulation of attention), since many children suffering from ADHD show electrophysiological hypoarousal [48]; this might impede the upregulation of attention but not its downregulation. Children on constant stimulant medication showed stronger learning across-sessions than children who did not take stimulants (irrespective of age), suggesting that MPH was a critical factor for substantial learning progress (i.e., in the deactivation task across-sessions).

#### 4.2.2. Within-Session Transfer Learning

Within sessions, transfer learning took place only in the activation task but remained unchanged in the deactivation task. Thus, subjects managed to improve the voluntary upregulation of attention within a session, while the voluntary downregulation of attention remained stable. It is difficult to interpret this finding. As with the within-session feedback learning, it might have taken the subjects some time to fully mobilize attentional resources within a session. Currently, no study on SCP-NF has reported results on within-session learning (but see 22, 24, 48, 49 for within-session analyses for frequency band NF in ADHD). Thus, further research is needed to map learning within sessions and to fully understand its interdependency with learning across sessions.

#### 4.2.3. General Discussion Transfer Learning

In transfer learning both within and across sessions, age was negatively associated with the mean amplitude irrespective of time or session number. This association was probably related to larger proportions of fast frequencies as a function of age [28–30]. One salient result is that cross-session transfer learning seems rather confined to the deactivation task, which we hypothesized to be a result of facilitated downregulation of attention due to hypoarousal, but within-session transfer learning is confined to the activation task, which we hypothesized to reflect a mobilization of attentional resources. Comparing these two different time windows reveals that learning does not take place in the activation and deactivation tasks concurrently, but across two different time windows (within and across sessions, resp.). Thus, considering both time windows provides a more complete picture of learning in NF than merely investigating cross-session learning, as is common. Learning to generate potential shifts without continuous performance feedback (transfer condition) is thought to be a better indicator for regulation capacities outside the laboratory than in situations where continuous performance feedback is available [10]. It is possible that more time might have been needed to practice the up- and downregulation of attention in the transfer condition. Thus, the ability to generalize the acquired skills might not have fully developed and might have needed more transfer training sessions.

### 4.3. General Discussion

Taken these findings together with respect to condition, task type, time window and subject-related or treatment-related factors, age and stimulants were the dominant moderators of learning: in medicated children, age was positively associated with NF performance while being negatively associated in nonmedicated children—for both within- and cross-session analyses. In contrast, transfer performance across time was only moderated by MPH and only when considered learning across sessions, but not within session. In this study, transfer and feedback trials were mixed within one block and the number of transfer trials increased across sessions.

#### 4.3.1. Effects Not Moderating Learning

Neither dosage nor duration of stimulant intake predicted learning. However, we cannot exclude any general effects of dosage and intake duration on learning, since dosage and duration of stimulant intake did not vary by amounts that might have led us to expect possible moderating effects. Clinical symptoms or severity rated by parents and teachers did not moderate learning. This was unexpected, since we had hypothesized a more severe initial impairment of attention to be reflected in weaker overall NF performance. However, clinical severity might have not been linearly associated with performance but might have been moderated by a threshold of relevant impairment; we did not investigate this issue. The artifact reduction rate has been shown in previous studies to improve over time, possibly as a nonspecific effect of the treatment helping children learn to sit still [12], but whether this reduction in artifacts is related to NF learning has rarely been examined. In the present study, inclusion of the artifact reduction did not result in a better model fit, which suggests that even though artifact reduction took place across sessions, it was not related to NF learning within or across sessions. Gender has rarely been included as a predictor due to the common overproportion of males in ADHD populations. As almost 50% of our participants were females, we could test for possible gender differences in NF learning. We might have included more females (almost 50%) than other studies because training took place not only in the clinic but also in schools, where we may have reached a more diverse population. However, including gender did not yield a better model fit.

It was not surprising that setting was not associated with NF learning, as NF learning should not be affected by the training environment; however, differential setting effects on NF learning have never been tested directly before, so our study is the first to provide empirical confirmation of this common assumption. Likewise, intersession interval has rarely been examined as an effect on NF learning or clinical improvement [49, 50]. It did not yield a better model fit here, but this might also be attributable to only small variations in the time schedule.

### 4.4. Mixed-Effects Modeling

By employing a mixed-effects modeling approach, we expected to achieve a more realistic mapping of NF learning in ADHD than other statistical models, such as multivariate analysis of variance (MANOVAs). First, results achieved by MANOVAs are very sensitive to outliers, and furthermore, results can easily be biased by unbalanced datasets and missing data. Mixed-effects models can deal with these impediments to a certain extent. A major advantage of our statistical approach when drawing conclusions about the usefulness of MPH for NF learning is that independence amongst observations is not a necessary precondition; performance variability can be accounted for both within a subject across sessions and between subjects. One limitation of this approach may be the lack of current consensus whether and if so by what degree it is possible to rely on *p* values in mixed modeling and on how to derive proper effect sizes (33).

### 4.5. Limitations

The study did not include follow-up or booster sessions. Although there is evidence that SCP-NF performance can be maintained at least up to two years [51], it would have been important to investigate whether we could have replicated these findings with a mixed-effects modeling approach. A more systematic study with randomization of children on and off medication would be needed to analyze this association and replicate our findings. In addition, this study did not include a NF control group to contrast learning effects that are characteristic for SCP-NF learning with other training protocols. We deliberately did not include clinical outcome data here to examine treatment efficacy. The aim of the paper was instead to present a novel methodological approach to the investigation of treatment moderators and treatment specificity.

### 4.6. Conclusion

Given the complex interactions in our results which have not been shown before, we conclude that mixed-effect modeling is an appropriate approach to analyze NF learning. We therefore suggest this approach for future research to reach a better understanding of the mechanism of NF learning and treatment specificity.

## Figures and Tables

**Table 1:** Description of participants.

	Total	With MPH	No MPH
*N* (total)	48	16	32
Male/female (*N*)	27/21	12/4	15/17
Clinical setting (*N*)	26	10	16
School setting (*N*)	22	6	16
Intersession interval (days)			
Clinic	4.1 ± 1.9	4.1 ± 8.3	4.4 ± 6.3
School	4.8 ± 1.1	4.8 ± 3.2	5.3 ± 7.7
Age (years) all	11.2 ± 2.2	10.9 ± 2.4	11.4 ± 2.0
MPH dosage (mg)	24.5 ± 15.1	23.6 ± 15.0	0
MPH intake duration (years)	2.3 ± 2.5	2.4 ± 2.5	0
Estimated IQ	109.5 ± 14.8	109.9 ± 14.7	109.1 ± 15.3
Clinical ratings before training			
DSM-IV C-3 P (T-scores)			
Inattention	67.8 ± 5.8	63.3 ± 6.4	67.5 ± 8.2
Hyperactivity/impulsivity	64.9 ± 8.4	66.1 ± 6.2	59.6 ± 9.1
DSM-IV C-3 T (T-scores)			
Inattention	65.7 ± 6.1	61.7 ± 7.0	64.2 ± 5.3
Hyperactivity/impulsivity	63.0 ± 7.9	59.96.9	62.9 ± 8.3
Clinical ADHD diagnosis (yes/no)	33	16/0	17/15

C-3 P/T: Conners 3 parent/teacher ratings (DSM-IV indices); MPH: methylphenidate.

**Table 2:** Effects considered for statistical analysis.

Model specifications	Measure
Time	
Cross-session model	Double session number (15 double sessions)
Within-session model	Bin number: 10 bins per session. The mean amplitude of baseline-corrected trials was averaged across sessions and then averaged across the ten equally spaced units (bins).
Condition type	
Feedback (FB)	Continuous performance feedback stimulus visible
Transfer (TR)	Performance feedback, delayed
*Effects*	
Tasks	Deactivation (generation of positive potential shifts of SCPs) versus activation (generation of negative potential shifts of SCPs)
Intersession interval	Days passed between training sessions
Age	In years (continuous variable in the model, only for visualization in plots dichotomized into younger and older age classes)
MPH	Being on constant stimulant medication (methylphenidate), factorized into yes versus no
Stimulants intake duration	Years of MPH intake
Dosage of stimulant medication	Methylphenidate (MPH) in mg
Sex	Factorized into female versus male
IQ	Estimated IQ (WISC-IV short form)
Setting	Factorized into school setting versus clinical setting
Severity of ADHD symptoms	*T* values of the Conners 3 DSM-IV indices for hyperactivity/impulsivity and inattention based on parent and teacher ratings before training
Preexisting ADHD diagnosis	Clinical ADHD diagnosis before entering the study factorized into yes versus no
Artifact rate	Percentage of rejected trials within a session

**Table 3:** Results for NF learning with respect to condition (feedback/transfer) and time (cross-/within-session).

	Cross-session learning						Within-session learning					
	Feedback			Transfer			Feedback			Transfer		
	B	CI	*p*	B	CI	*p*	B	CI	*p*	B	CI	*p*
*Fixed parts*												
Intercept	−3.95	−5.58 to −2.44	**<0.001**	−1.06	−2.72–0.68	0.218	−2.65	−3.86 to −1.40	**<0.001**	1.27	−0.38–2. 68	0.095
Session	−0.00	−0.17–0.16	0.994	0.03	−0.18–0.21	0.797						
Bins							−0.29	−0.40 to −0.18	**<0.001**	−0.40	−0.61 to −0.20	**<0.001**
Task	1.66	−0.04–3.31	0.061	−2.40	−4.50 to −0.56	**0.014**	2.79	2.14–3.41	**<0.001**	−2.92	−4.34 to −1.27	**<0.001**
Age	−0.49	−1.28–0.27	0.214	−0.59	−0.94 to −0.24	**0.002**	−0.62	−1.20 to −0.16	**0.014**	−0.40	−0.78 to −0.00	**0.040**
MPH	1.18	−1.44–3.66	0.384	4.11	1.34–7.13	**0.006**	1.17	−0.57–2.87	0.176	1.71	−0.03–3.40	0.051
IQ	−0.08	−0.14 to −0.02	**0.006**				−0.07	−0.13 to −0.01	**0.018**			
Session: task	0.15	−0.02–0.34	0.123	0.20	−0.02–0.4	0.072						
Bins: task										0.33	0.07–0.56	**0.011**
Session: age	−0.01	−0.09–0.08	0.858									
Task: age	−0.42	−1.33–0.43	0.342				−0.40	−0.71 to −0.08	**0.016**			
Session: MPH	−0.05	−0.32–0.21	0.745	−0.27	−0.63–0.06	0.124						
Task: MPH	−1.56	−4.53–1.91	0.308	−4.35	−7.54 to −1.13	**0.010**	−0.79	−1.98–0.39	0.177			
Age: MPH	0.38	−4.53–1.91	0.538				−0.73	−1.45–0.09	0.066			
Session: task: age	−0.01	−0.12–0.09	0.804									
Session: task: MPH	0.12	−0.20–0.46	0.464	0.39	0.05–0.76	**0.036**						
Session: age: MPH	−0.15	−0.28 to −0.01	**0.029**									
Task: age: MPH	−1.41	−2.70–0.019	**0.042**				0.86	0.36–1.35	**0.001**			
Session: task: age: MPH	0.32	0.16–0.47	**<0.001**									
*Random parts*												
*σ* ^2^	41.776			49.174			17.870			32.477		
*τ*_00, subject_	7.214			8.501			9.093			13.320		
*τ*_11, session_	0.0734			0.1325								
*τ*_11, bins_							0.05569			0.1231		
*ρ*_01_	−0.455			−0.620			−0.585			−0.735		
Observations	1400			1380			959			959		

Mixed-effects model results for NF learning. The dependent variable is mean amplitude (*μ*V) of baseline-corrected trials. Feedback/transfer: condition where a feedback stimulus is (feedback) or is not (transfer) visible. Session: session number, 15 double training sessions in total. Bins: bin number, 10 bins in total. Task: performance in the deactivation (generation of positive potential shifts) versus activation tasks (generation of negative potential shifts). MPH: on constant methylphenidate medication (yes versus no). *α*2: within-subject residual variance. *τ*_00_, subject: between-subject variance. *τ*_11_: random slope variance. *ρ*_01_: random intercept slope correlation.

## Supplementary Materials

Supplementary Materials Figure S1: visualization of cross-session NF learning in the feedback (A) and transfer conditions (B). The dependent variable is the difference between mean amplitude (visualization of cross-session NF learning in the feedback (A) and transfer conditions (B)). For raw data, see scatter plot under each effects panel, fitted with a fixed linear regression based on the same factors as in A. A: interaction plot for the fixed effects session number, MPH, and age. B: interaction plot for the fixed effects session number and MPH. Session number: 15 sessions in total. Condition: deactivation: generation of positive potential shifts. Activation: generation of negative potential shifts. MPH: being on regular methylphenidate medication (yes versus no). Condition: feedback: feedback stimulus visible. Transfer: no feedback stimulus visible. A and B: for visualization age is subdivided into two age classes (8–12 and 13–16 years), but preserved as a continuous variable in the original model. Table S2: results for linear mixed effects models for cross-session NF learning with a condition type (feedback/transfer) in titles. Table S3: ANOVA results of models predicting NF performance in the feedback condition. Table S4: ANOVA results of models predicting NF performance in the transfer condition.
