# Supplementary material for: Mixed-Effects Modeling of Neurofeedback Self-Regulation Performance: Moderators for Learning in Children with ADHD
Source: Neural Plast. 2018 Mar 22;2018:2464310. doi: 10.1155/2018/2464310 (PMC5885491; doi:10.1155/2018/2464310)
Supplement: Supplementary Materials — Figure S1: visualization of cross-session NF learning in the feedback (A) and transfer conditions (B). The dependent variable is the difference between mean amplitude (visualization of cross-session NF learning in the feedback (A) and transfer conditions (B)). For raw data, see scatter plot under each effects panel, fitted with a fixed linear regression based on the same factors as in A. A: interaction plot for the fixed effects session number, MPH, and age. B: interaction plot for the fixed effects session number and MPH. Session number: 15 sessions in total. Condition: deactivation: generation of positive potential shifts. Activation: generation of negative potential shifts. MPH: being on regular methylphenidate medication (yes versus no). Condition: feedback: feedback stimulus visible. Transfer: no feedback stimulus visible. A and B: for visualization age is subdivided into two age classes (8–12 and 13–16 years), but preserved as a continuous variable in the original model. Table S2: results for linear mixed effects models for cross-session NF learning with a condition type (feedback/transfer) in titles. Table S3: ANOVA results of models predicting NF performance in the feedback condition. Table S4: ANOVA results of models predicting NF performance in the transfer condition. [file 2464310.f1.docx]

Supplement

B

A


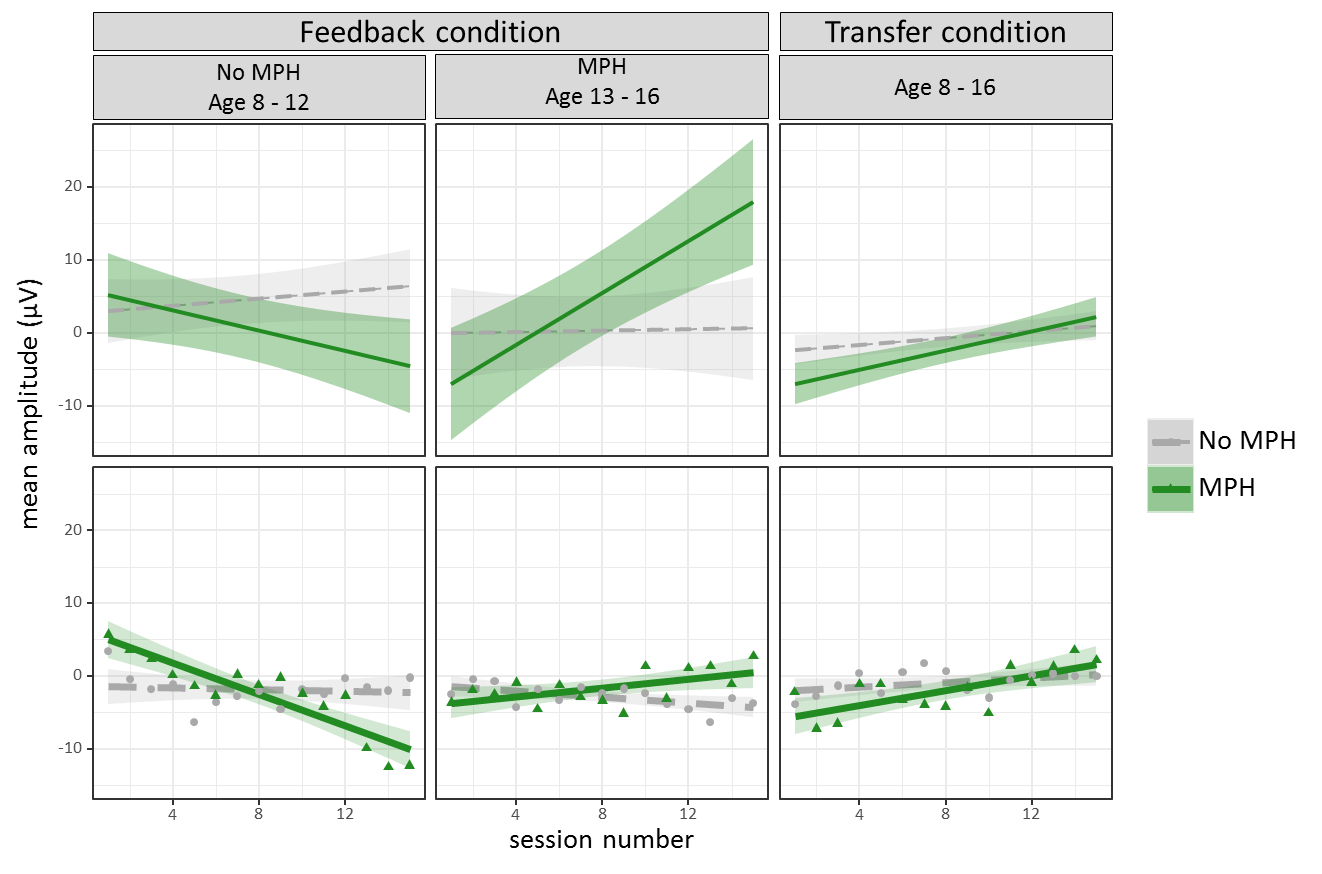


*Figure S1.* Visualization of cross- session NF-learning in the Feedback (A) and Transfer condition (B). The dependent variable is the difference between mean amplitude (visualization of cross-session NF-learning in the Feedback (A) and Transfer condition (B). For raw data, see scatter plot under each effects panel, fitted with a fixed linear regression based on the same factors as in A. **A**: Interaction plot for the fixed effects session number, MPH and age. **B**: Interaction plot for the fixed effects session number and MPH. *Session number:* 15 sessions in total. *Condition:* Deactivation: Generation of positive potential shifts. Activation: Generation of negative potential shifts. *MPH:* being on regular methylphenidate medication (yes versus no). *Condition:* Feedback: Feedback stimulus visible. Transfer: No feedback stimulus visible. **A and B**: For visualization age is subdivided into two age classes (8-12 and 13-16 years), but preserved as a continuous variable in the original model.

*Table S2*

*Results for linear mixed effects models for cross-session NF learning with condition type (Feedback / Transfer) in titles.*


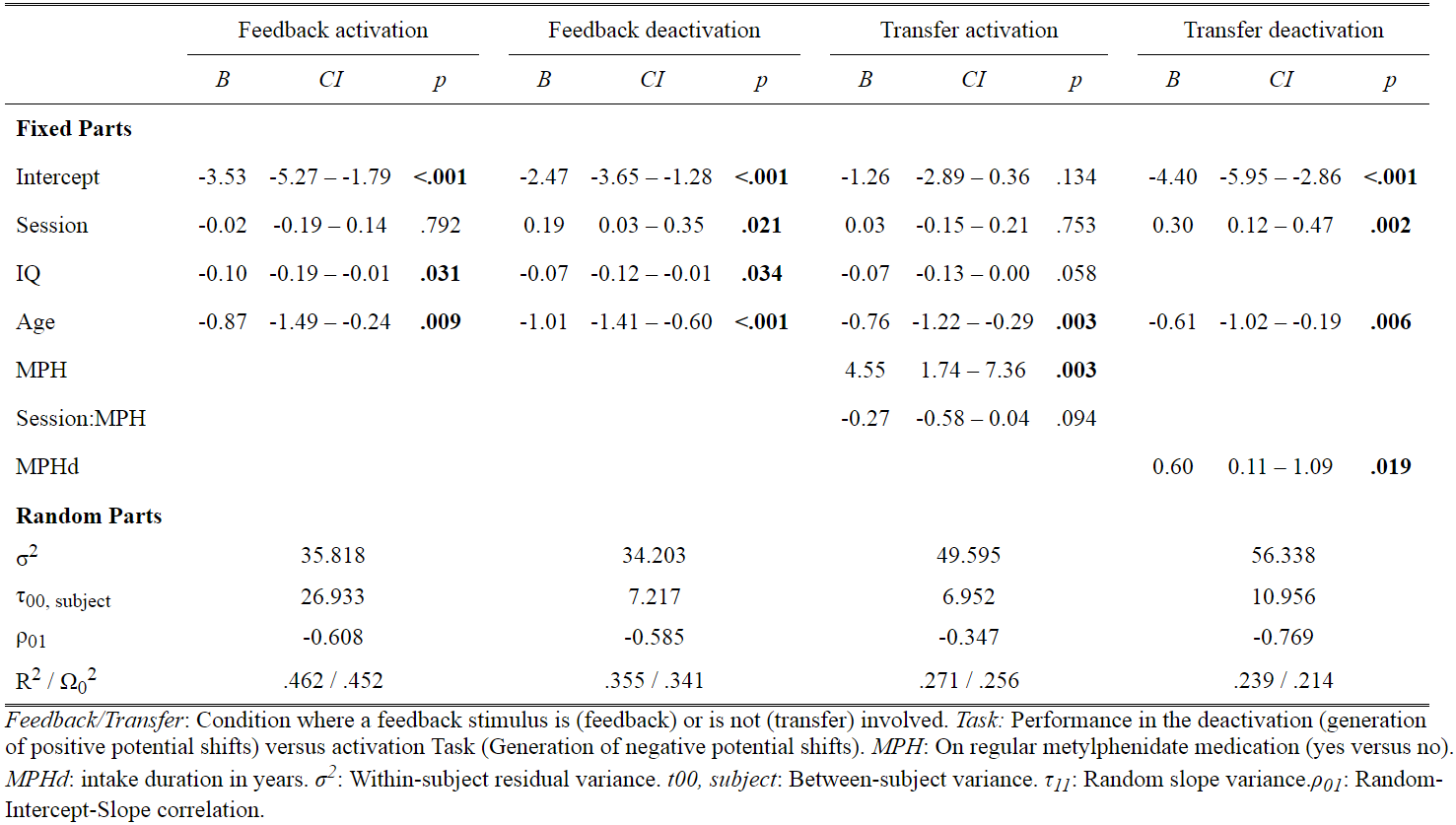


Table S3.

ANOVA results of models predicting NF performance in the feedback condition.

|  | Cross session feedback learning | | | | Within session feedback Performance | | | |
| --- | --- | --- | --- | --- | --- | --- | --- | --- |
|  | Df | Sum Sq | Mean Sq | F value | Df | Sum Sq | Mean Sq | F value |
| Session | 1 | 99.58 | 99.58 | 2.36 |  |  |  |  |
| Bins |  |  |  |  | 1 | 370.32 | 370.32 | 20.91 |
| Task | 1 | 2497.21 | 2497.21 | 59.18 | 1 | 1483.09 | 1483.09 | 83.73 |
| Age | 1 | 280.68 | 280.681 | 6.65 | 1 | 105.45 | 105.45 | 5.95 |
| MPH | 1 | 65.65 | 65.65 | 1.55 | 1 | 42.01 | 42.01 | 2.37 |
| IQ | 1 | 435.33 | 435.33 | 10.32 | 1 | 189.38 | 189.38 | 10.69 |
| Session : Task | 1 | 241.64 | 241.64 | 5.73 |  |  |  |  |
| Session : Age | 1 | 1.50 | 1.50 | 0.04 | 1 | 10.30 | 10.30 | 0.58 |
| Task : Age | 1 | 10.49 | 10.49 | 0.25 |  |  |  |  |
| Session : MPH | 1 | 2.93 | 2.93 | 0.07 |  |  |  |  |
| Task : MPH | 1 | 32.32 | 32.32 | 0.77 | 1 | 50.76 | 50.76 | 2.87 |
| Age : MPH | 1 | 0.90 | 0.902 | 0.02 | 1 | 0.20 | 0.20 | 0.01 |
| Session : Task : Age | 1 | 378.73 | 378.73 | 8.98 |  |  |  |  |
| Session : Task : MPH | 1 | 15.40 | 15.40 | 0.36 |  |  |  |  |
| Session : Age : MPH | 1 | 5.70 | 5.70 | 0.14 |  |  |  |  |
| Task : Age : MPH | 1 | 437.30 | 437.39 | 10.36 |  |  |  |  |
| Session : Task : Age : MPH | 1 | 635.24 | 635.24 | 15.05 | 1 | 158.53 | 158.53 | 8.95 |

*Note.* The dependent variable is mean amplitude (difference measure for mean amplitude per session of the positivity task minus the mean amplitude of the negativity task per session). The best model fit was yielded by a four-way interaction between session, task, age and MPH affecting the dependent variable mean amplitude. Significance level was p<0.05.

Table S4.

ANOVA results of models predicting NF performance in the transfer condition.

|  | Cross session Feedback Performance | | | | Within Session Transfer Performance | | | |
| --- | --- | --- | --- | --- | --- | --- | --- | --- |
|  | Df | Sum Sq | Mean Sq | F value | Df | Sum Sq | Mean Sq | F value |
| Session number | 1 | 129.49 | 129.49 | 2.57 |  |  |  |  |
| Bin number |  |  |  |  | 1 | 370.32 | 370.32 | 20.91 |
| Task | 1 | 615.04 | 615.04 | 12.22 |  |  |  |  |
| Age | 1 | 447.30 | 447.31 | 8.89 | 1 | 1483.09 | 1483.09 | 83.74 |
| MPH | 1 | 126.30 | 126.31 | 2.51 | 1 | 42.01 | 42.01 | 2.37 |
| IQ |  |  |  |  | 1 | 189.38 | 189.38 | 10.69 |
| Session : Task | 1 | 687.96 | 687.96 | 13.66 |  |  |  |  |
| Session : Age |  |  |  |  |  |  |  |  |
| Task : Age |  |  |  |  | 1 | 10.30 | 10.30 | 0.58 |
| Session : MPH | 1 | 2.94 | 2.94 | 0.06 |  |  |  |  |
| Task : MPH | 1 | 220.98 | 220.98 | 4.39 | 1 | 50.76 | 50.76 | 2.87 |
| Age : MPH |  |  |  |  | 1 | 0.20 | 0.20 | 0.01 |
| Session : Task : Age |  |  |  |  |  |  |  |  |
| Session : Task : MPH | 1 | 271.03 | 271.03 | 5.38 |  |  |  |  |
| Session : Age : MPH |  |  |  |  |  |  |  |  |
| Task : Age : MPH |  |  |  |  | 1 | 158.53 | 158.53 | 8.95 |
| Session : Task : Age : MPH |  |  |  |  |  |  |  |  |

*Note.* The dependent variable is mean amplitude (difference measure for mean amplitude per bin of the positivity task minus the mean amplitude of the negativity task per bin). The best model fit was yielded by a four-way interaction between session, task, age and MPH affecting the dependent variable mean amplitude. Significance level was p<0.05.
